# Immunization against the Spread of Rumors in Homogenous Networks

**DOI:** 10.1371/journal.pone.0124978

**Published:** 2015-05-01

**Authors:** Laijun Zhao, Jiajia Wang, Rongbing Huang

**Affiliations:** 1 Sino-US Global Logistics Institute, Shanghai Jiao Tong University, Shanghai 200030, P.R. China; 2 Antai College of Economics & Management, Shanghai Jiao Tong University, Shanghai 200052, P.R. China; 3 School of Administrative Studies, York University, Toronto ON M3J 1P3, Canada; Cinvestav-Merida, MEXICO

## Abstract

Since most rumors are harmful, how to control the spread of such rumors is important. In this paper, we studied the process of "immunization" against rumors by modeling the process of rumor spreading and changing the termination mechanism for the spread of rumors to make the model more realistic. We derived mean-field equations to describe the dynamics of the rumor spread. By carrying out steady-state analysis, we derived the spreading threshold value that must be exceeded for the rumor to spread. We further discuss a possible strategy for immunization against rumors and obtain an immunization threshold value that represents the minimum level required to stop the rumor from spreading. Numerical simulations revealed that the average degree of the network and parameters of transformation probability significantly influence the spread of rumors. More importantly, the simulations revealed that immunizing a higher proportion of individuals is not necessarily better because of the waste of resources and the generation of unnecessary information. So the optimal immunization rate should be the immunization threshold.

## Introduction

Rumors represent unproven expositions about or interpretations of news, events, or problems that are of public interest. Because rumors are unconfirmed information, it is hard to determine whether they are true or false. However, many case studies indicate that most rumors are false news, which exert negative impacts on the public and on social security [1, 2]. In particular, the rapid development of Internet information technology has let rumors spread rapidly through new media, thereby influencing many aspects of our daily life [3, 4]. For example, after the Fukushima Daiichi nuclear disaster in Japan in 2011, which was initiated by an earthquake and tsunami, the Chinese public began panic buying of iodized salt due to rumors that the ingestion of salt containing iodine could prevent radiation damage and that the leakage of radioactive material had led to pollution of the sea, thereby decreasing the safety of sea salt. Supermarkets sold out of iodized salt, and many businesses seized the chance to raise the price of iodized salt, which lead to public disturbances [5]. Another example comprises the doomsday rumors in 2012. On December 14, 2012, a man in Guangshan County of China’s Henan Province injured 23 innocent pupils in a primary school, apparently because he believed that the world was ending [6]. Because of dangerous consequences, the need to reduce the spread of rumors and to weaken their potential harm has become increasingly important in China. Currently, the Chinese government has made it illegal to create and disseminate false information through the Internet. However, most of the illegal rumor-passing behaviors have relatively low costs for the creators and spreaders of rumors, and many illegal behaviors are never detected or prosecuted; thus, rumor spread is not significantly deterred.

Scholars look for rumor-spread laws through establishing rumor-spread models. A rumor is analogous to a virus in the way that it spreads among individuals, so existing rumor-spread models are mostly inspired by the research results of infectious disease models [7–10]. The first rumor-spread mathematical model was proposed by Daley and Kendall in 1965, called DK model [11, 12]. In the DK model, the crowd was divided into three categories: people who do not know the rumor, people who know and propagate the rumor, and people who know the rumor but do not transmit it. Rumors spreading from the spreader to others through a two-way communication link, following the mass action law. Maki and Thompson [13] thought that when a spreader contacts with another spreader, only the first one stop propagating the rumor. Based on this, they established the MT model in 1973. The above classical rumor-spread models laid a foundation for the follow-up studies. However, Sudbury [14] considered that the DK and MT models do not account for networks’ topological characteristics and are not suitable for description of the large-scale rumors’ spreading process. He also suggested that the dynamic behaviors of rumor spreading matched the SIR epidemic model, in which “S”, “I”, and “R” correspond (respectively) to susceptible individuals, infected individuals, and removed individuals. Zanette [15, 16] firstly applied complex network theory to the rumors-spread researches. He simplified the rumor-spread mechanism, and built a rumor-spread model in a small-world network. Moreno et al. [17] standardized the crowd classification and developed SIR rumor spreading model both in homogenous networks like small-world networks which have the exponential degree distribution and in heterogeneous networks like scale-free networks which have the power-law degree distribution. They used “Ignorant” (I), “Spreader” (S) and “Stifler” (R) to represent the individuals who have no idea about the rumor, who know and propagate the rumor and who know the rumor but do not transmit it, respectively. So the later rumor-spread models followed the classification representation method of “Ignorant”, “Spreader”, and “Stifler”. Nekovee et al.[18] regarded forgetting as a very important factor of rumor termination. Therefore, they introduced a forgetting mechanism into the SIR rumor spreading model and derived the mean-field equations in complex networks. They also confirmed the existence of a critical threshold for the rumor spreading in complex networks. On the basis of [18], Zhao et al.[19, 20] not only considered the forgetting mechanism, but also considered the remembering mechanism and investigated the resulting dynamics of rumor spreading in complex networks. One assumption of rumors termination in the above models is that when a spreader contacts another spreader, the former one may become a stifler because he/she believes the rumor is outdated information. However, in real life it is possible that the initiating spreader found other people also spread the rumor and the rumor-spread may be a fashion. So this can inspire the enthusiasm of the rumor-spread instead of stop propagating.

Understanding the rumor propagation laws is to provide more effective means of controlling the spread of harmful rumors. At present, there are two kinds of quantitative methods to prevent and control the rumor propagation: one is through changing the networks’ topology characteristics of rumor-spread. For example, Pan et al. [21] analyzed the spread of rumors in scale-free networks with variable clustering coefficients. They found that higher clustering coefficients inhibited the transmission of rumors, whereas lower clustering coefficients increased the spread of rumors. Another is through external intervention strategies. It is necessary to implement immunization to ignorant individuals, just like those on epidemic models [22–25]. In the rumor-spread models, there are two kinds of immunization strategies. One is random immunization strategy that randomly select some percentage of the individuals and let them learn the falsity of the rumor. The other is targeted immunization that selectively choose a few key individuals and let them know the truth of the rumor. Huang and Jin [26] applied random immunization and targeted immunization to rumor spreading. They found that both immunization strategies are effective and that the immunization effect was strongly related to the average degree of the network. Singh and Singh [27] noted that a given node in a network may contact some but not all of its neighbors, which modifies how the rumor can spread. They therefore modified the SIR rumor model to account for this dependence of the rate of spread, after which they obtained thresholds for spreading under the assumptions of random and targeted immunizations. Their results showed that targeted immunization is more effective than random immunization in scale-free networks. Inspired by them, we can apply the immunization to our rumor-spread model.

In this paper, we modified a SIR rumor-spread model and provided a kind of method to suppress the propagation of harmful rumors. First, we refine this model and establish mean-field equations to describe the spreading process. Next, we discuss the spreading threshold for steady-state spread of a rumor, and derive an immunization threshold under the rumor immunization strategy. Finally, we perform numerical simulations to examine the dynamics of the modified model. We examine how the spread of rumors can be controlled by changing parameters and immunizing certain amount of nodes.

## The rumor-spread model

**Fig 1:**
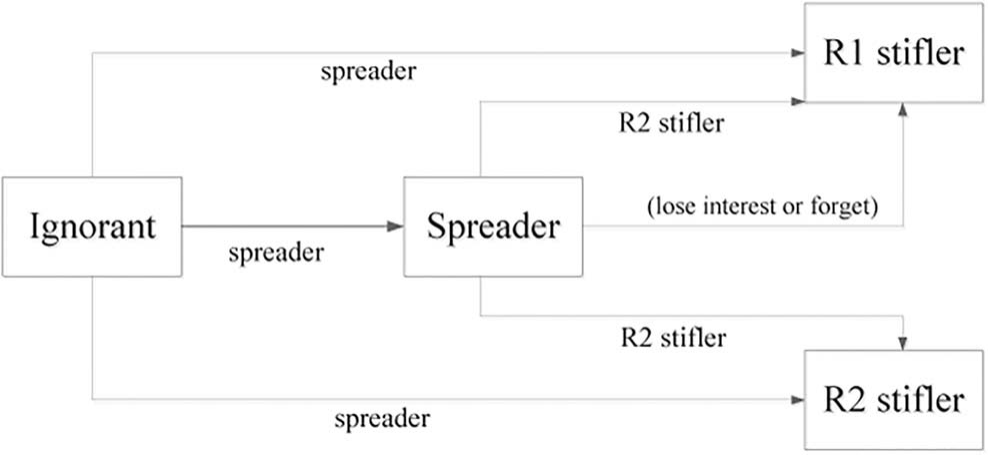
Structure of the modified SIR rumor spreading model for the rumor-spread process. R1 represents the stiflers who do not know the truth or falsehood of the rumor and simply lose interest in spreading the rumor, and R2 represents stiflers who see the falsity of the rumor and oppose its spread. The sub-script line label indicates the interaction partner, the super-script line label indicates the rate of state transition.

As mentioned above, in the SIR rumor spreading model, the total population was divided into three groups: ignorants, spreaders, and stiflers. Assuming that when an ignorant contacts a spreader, the ignorant is susceptible to be informed and to become a spreader with a certain probability. When a spreader contacts another spreader or a stifler, the initiating spreader may no longer spread the rumor and become a stifler with a certain probability. To produce a more realistic model that supports an immunization strategy, we divided the stiflers into two groups: one is the population who do not know the truth or falsehood of the rumor and who simply lose interest in spreading the rumor; the other is the population who see the falsity of the rumor and oppose its spread, so they can be regarded as immunized individuals. We assumed that a closed and mixed population consisting of *N* individuals would form a homogeneous network and that the rumor is disseminated through direct contacts between these individuals. The rumor-spread process in this model is shown in Fig 1.

The rules of propagation are as follows: When an ignorant contacts a spreader, the ignorant may become a spreader with probability *λ*, an R1 stifler with probability *γ*, or an R2 stifler with probability *β*. Because the ignorant cannot be unaware of the rumor once they hear it, we assume that *λ* + *β* + *γ* = 1. When a spreader contacts an R2 stifler, the spreader may turn into an R1 stifler with probability *α* or an R2 stifler with probability *θ*, and *α* + *θ* ≤ 1. In addition, a spreader can spontaneously stop spreading the rumor and become an R1 stifler who forgets or loses interest in spreading the rumor, with probability *δ*. The most significant difference between this modified model and the original model is the mechanism that stops spreading of the rumor, since individuals probably stop spreading a rumor not because they know that the rumor is outdated, but rather because they figure out that the rumor is wrong or lose interest in spreading the rumor. Moreover, *I*(*t*), *S*(*t*), *R*1(*t*), and *R*2(*t*) denote the density of these four types of individuals at time *t*, respectively. In this case, according to the rumor-spread process elaborated earlier in this section, the mean-field equations can be presented as follows:

$$\begin{array}{ccc} \frac{dI(t)}{dt} & = & -\bar{k}I\left(t\right)S\left(t\right) \end{array}$$ (1)

$$\begin{array}{ccc} \frac{dS(t)}{dt} & = & \lambda \bar{k}I\left(t\right)S\left(t\right)-\alpha \bar{k}S\left(t\right)R2\left(t\right)-\theta \bar{k}S\left(t\right)R2\left(t\right)-\delta S\left(t\right) \end{array}$$ (2)

$$\begin{array}{ccc} \frac{dR1(t)}{dt} & = & \gamma \bar{k}I\left(t\right)S\left(t\right)+\alpha \bar{k}S\left(t\right)R2\left(t\right)+\delta S\left(t\right) \end{array}$$ (3)

$$\begin{array}{ccc} \frac{dR2(t)}{dt} & = & \beta \bar{k}I\left(t\right)S\left(t\right)+\theta \bar{k}S\left(t\right)R2\left(t\right) \end{array}$$ (4)

where $\bar{k}$ denotes the average degree of the network. The normalization condition is *I*(*t*) + *S*(*t*) + *R*1(*t*) + *R*2(*t*) = 1.

## Steady-state analysis

The spreading process starts with a few people being informed of a rumor, so we assumed that the initial condition for spreading of the rumor is $S(0)=\frac{1}{N}\approx 0,I(0)=\frac{N-1}{N}\approx 1$ and *R*1(0) = *R*2(0) = 0. We can then divide Eq (3) and Eq (4) by Eq (1) to obtain the following results:

$$\begin{array}{ccc} \frac{dR1(t)}{dI(t)} & = & \frac{\gamma \bar{k}I\left(t\right)S\left(t\right)+\alpha \bar{k}S\left(t\right)R2\left(t\right)+\delta S\left(t\right)}{-\bar{k}I\left(t\right)S\left(t\right)}=-\gamma -\alpha \frac{R2(t)}{I(t)}-\frac{\delta }{\bar{k}I\left(t\right)}\\ \frac{dR2(t)}{dI(t)} & = & \frac{\beta \bar{k}I\left(t\right)S\left(t\right)+\theta \bar{k}S\left(t\right)R2\left(t\right)}{-\bar{k}I\left(t\right)S\left(t\right)}=-\beta -\theta \frac{R2(t)}{I(t)} \end{array}$$

Given *x* = *I*(*t*), *y* = *R*1(*t*), and *z* = *R*2(*t*), then:

$$\begin{array}{ccc} \frac{dy}{dx} & = & -\gamma -\alpha \frac{z}{x}-\frac{\delta }{\bar{k}x} \end{array}$$ (5)

$$\begin{array}{ccc} \frac{dz}{dx} & = & -\beta -\theta \frac{z}{x} \end{array}$$ (6)

First, we calculate the value of Eq (6). Let *z*/*x* = *u*, so *dz* = *xdu* + *udx*, and:

$$\begin{array}{c} \frac{dz}{dx}=\frac{xdu+udx}{dx}=-\beta -\theta u \end{array}$$

$$\begin{array}{ccc} ∴ & & xdu+udx=-\beta dx-\theta udx\\ & \Rightarrow & -\frac{1}{\theta +1}\ln\left(\beta +\theta u+u\right)=\ln C_{1}x\\ & \Rightarrow & u=-\frac{\beta }{\theta +1}+\frac{C_{2}x^{-(\theta +1)}}{\theta +1},C_{2}=C_{1}^{-(\theta +1)} \end{array}$$

$$\begin{array}{ccc} ∴ & & z=-\frac{\beta }{\theta +1}x+\frac{C_{2}}{\theta +1}x^{-\theta }\\ & \Rightarrow & R2\left(t\right)=-\frac{\beta }{\theta +1}I\left(t\right)+\frac{C_{2}}{\theta +1}I{\left(t\right)}^{-\theta } \end{array}$$ (7)

Considering the initial condition *I*(0) ≈ 1, *R*2(0) = 0, we obtain:

$$\begin{array}{ccc} & & 0=-\frac{\beta }{\theta +1}+\frac{C_{2}}{\theta +1}\\ & \Rightarrow & C_{2}=\beta \end{array}$$

$$\begin{array}{c} ∴R2\left(t\right)=-\frac{\beta }{\theta +1}I\left(t\right)+\frac{\beta }{\theta +1}I{\left(t\right)}^{-\theta } \end{array}$$ (8)

that is:

$$\begin{array}{c} z=-\frac{\beta }{\theta +1}x+\frac{\beta }{\theta +1}x^{-\theta } \end{array}$$ (9)

We plug Eq (9) into Eq (5) and obtain:

$$\begin{array}{ccc} & & \frac{dy}{dx}=-\gamma +\frac{\alpha \beta }{\theta +1}-\frac{\alpha \beta }{\theta +1}x^{-(\theta +1)}-\frac{\delta }{\bar{k}x}\\ & \Rightarrow & y=\left(-\gamma +\frac{\alpha \beta }{\theta +1}\right)x+\frac{\alpha \beta }{\theta (\theta +1)}x^{-\theta }-\frac{\delta }{\bar{k}}\ln x+C_{3}\\ & \Rightarrow & R1\left(t\right)=\left(-\gamma +\frac{\alpha \beta }{\theta +1}\right)I\left(t\right)+\frac{\alpha \beta }{\theta (\theta +1)}I{\left(t\right)}^{-\theta }-\frac{\delta }{\bar{k}}\ln I\left(t\right)+C_{3} \end{array}$$

Considering the initial condition *I*(0) ≈ 1, *R*1(0) = 0, we have:

$$\begin{array}{ccc} & & 0=-\gamma +\frac{\alpha \beta }{\theta +1}+\frac{\alpha \beta }{\theta (\theta +1)}+C_{3}\\ & \Rightarrow & C_{3}=\frac{\gamma \theta -\alpha \beta }{\theta } \end{array}$$

$$\begin{array}{c} ∴R1\left(t\right)=\left(-\gamma +\frac{\alpha \beta }{\theta +1}\right)I\left(t\right)+\frac{\alpha \beta }{\theta (\theta +1)}I{\left(t\right)}^{-\theta }-\frac{\delta }{\bar{k}}\ln I\left(t\right)+\frac{\gamma \theta -\alpha \beta }{\theta } \end{array}$$ (10)

Let us define *I* = *I*(∞) = lim_*t*→∞_ *I*(*t*), *R*1 = *R*1(∞) = lim_*t*→∞_ *R*1(*t*), *R*2 = *R*2(∞) = lim_*t*→∞_ *R*2(*t*), and *R* = *R*1 + *R*2. *R* is the final size of the rumor, which represents the level of the rumor’s influence. In the final equilibrium state, there are ignorants, R1 stiflers, and R2 stiflers left in the system. Therefore:

$$\begin{array}{c} I=\lim_{t\to \infty }I\left(t\right)=1-\lim_{t\to \infty }R1\left(t\right)-\lim_{t\to \infty }R2\left(t\right)=1-R1-R2=1-R \end{array}$$

So:

$$\begin{array}{ccc} & & I=1-\left(\frac{\alpha \beta }{\theta +1}-\gamma \right)I-\frac{\alpha \beta }{\theta (\theta +1)}I^{-\theta }+\frac{\delta }{\bar{k}}\ln I-\frac{\gamma \theta -\alpha \beta }{\theta }+\frac{\beta }{\theta +1}I-\frac{\beta }{\theta +1}I^{-\theta }\\ & \Rightarrow & R=\frac{-\theta \beta -\alpha \beta }{\theta (\theta +1)}-\frac{\beta (\alpha -1)-\gamma (\theta +1)}{\theta +1}R+\frac{\alpha \beta +\theta \beta }{\theta (\theta +1)}{\left(1-R\right)}^{-\theta }-\frac{\delta }{\bar{k}}\ln\left(1-R\right) \end{array}$$

Given:

$$\begin{array}{ccc} f(R) & = & R+\frac{\theta \beta +\alpha \beta }{\theta (\theta +1)}+\frac{\beta (\alpha -1)-\gamma (\theta +1)}{\theta +1}R-\frac{\alpha \beta +\theta \beta }{\theta (\theta +1)}{\left(1-R\right)}^{-\theta }+\frac{\delta }{\bar{k}}\ln\left(1-R\right) \end{array}$$

Taking the derivative of *f*(*R*) with respect to *R*, we have:

$$\begin{array}{ccc} f\prime \left(R\right) & = & 1-\gamma +\frac{\beta (\alpha -1)}{\theta +1}-\frac{\alpha \beta +\theta \beta }{\theta +1}{\left(1-R\right)}^{-\theta -1}-\frac{\delta }{\bar{k}}\frac{1}{1-R}\\ f\prime \prime \left(R\right) & = & -\left(\alpha \beta +\theta \beta \right){\left(1-R\right)}^{-\theta -2}-\frac{\delta }{\bar{k}}{\left(1-R\right)}^{-2}\\ ∵ & & -\left(\alpha \beta +\theta \beta \right)<0,-\frac{\delta }{\bar{k}}<0\\ ∴ & & f\prime \prime \left(R\right)<0 \end{array}$$

That is, *f*(*R*) is a concave function on the interval [0, 1). Also:

$$\begin{array}{c} f\left(0\right)=-\frac{\alpha \beta +\theta \beta }{\theta (\theta +1)}+\frac{\theta \beta +\alpha \beta }{\theta (\theta +1)}=0 \end{array}$$

$$\begin{array}{ccc} f\prime \left(0\right) & = & 1-\gamma +\frac{\beta (\alpha -1)}{\theta +1}-\frac{\alpha \beta +\theta \beta }{\theta +1}-\frac{\delta }{\bar{k}}\\ & = & \lambda -\frac{\delta }{\bar{k}} \end{array}$$

$$\begin{array}{ccc} \lim_{R\to 1-}f\left(R\right) & = & \lim_{R\to 1-}\left[\left(1-\gamma +\frac{\beta \alpha -\beta }{\theta +1}\right)R-\frac{\alpha \beta +\theta \beta }{\theta (\theta +1)}{\left(1-R\right)}^{-\theta }+\frac{\delta }{\bar{k}}\ln\left(1-R\right)+\frac{\theta \beta +\alpha \beta }{\theta (\theta +1)}\right]\\ & = & 1-\gamma +\frac{\alpha \beta }{\theta }-\infty -\infty \\ & = & -\infty <0 \end{array}$$

**Fig 2:**
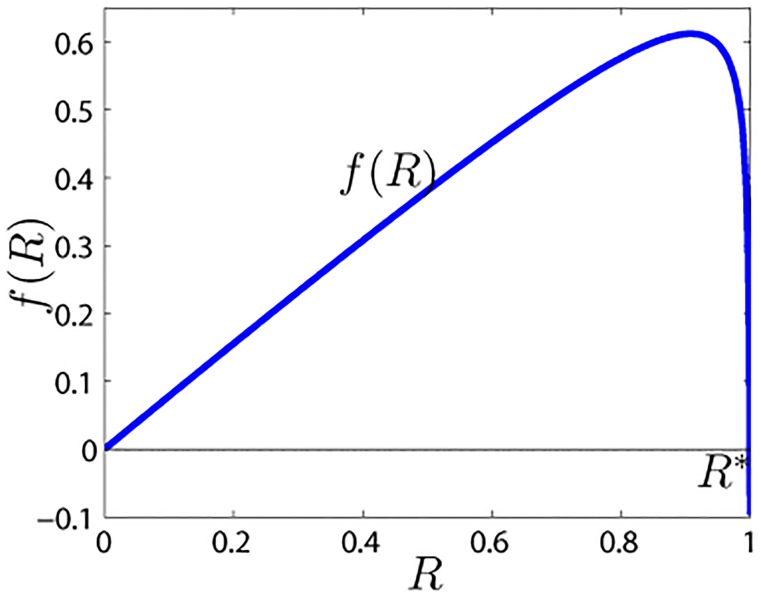
Shape of the function *f*(*R*). The parameters are *β* = 0.03, *γ* = 0.15, *θ* = 0.45, *α* = 0.25, *δ* = 0.35, and $\bar{k}=10$.

So, when *f*′(0) > 0 (i.e., $\lambda >\frac{\delta }{\bar{k}}$), there exists an R* ∈ (0, 1) satisfying *f*(R*) = 0 (Fig 2). In other words, we can obtain the spreading threshold $\lambda_{c}=\frac{\delta }{\bar{k}}$ in the modified rumor model.

If the rumor-spread rate *λ* is less than or equal to the rumor-spread threshold *λ*_*c*_ the rumor will not spread. Then we can define two ways to prevent the spread of rumors. The first is to decrease the probability that the rumor will spread (*λ*), which requires every individual in society to develop the habit of rational conversation and discussion that leads them to determine the authenticity of rumors through the process of evidence analysis, logical analysis, and common-sense analysis. It is also necessary for each individual to summarize the evidence and make their own decision about the truth or falsity of the rumor (i.e., to self-summarize), and individuals must also take precautions against spreading rumors. To accomplish this first method, it is necessary to raise the capabilities of rational analysis to detect hearsay. The second approach is to increase the rumor-spread threshold (*λ*_*c*_). Based on $\lambda_{c}=\frac{\delta }{\bar{k}}$, we can see that a high *δ* and a low $\bar{k}$ will increase *λ*_*c*_. Distracting potential rumor spreaders by revealing more interesting information can increase *λ*_*c*_ and decrease the risk of a rumor spreading. For instance, on 20 June 2011, a Chinese woman named Meimei Guo flaunted her luxurious life on the Web and declared that she was the commercial general manager of China’s Red Cross organization, leading to an uproar. But on 23 July 2011, news networks reported that two high-speed trains traveling on the Yongtaiwen railway line had collided on a bridge in the suburbs of Wenzhou, in China’s Zhejiang Province. The train collision quickly distracted public attention from Guo, who was soon forgotten by the public. To decrease $\bar{k}$, we can consider the example of new media, such as the popularity of Facebook and other social networks, in which the network’s average degree tends to become larger and rumor spreading becomes easier than ever before; thus, rumors may exert much larger influence than in the past. Thus, controlling the spread of rumors through homogenous networks may reduce the network’s average degree, thereby increasing the spreading threshold.

## Rumor immunization

Next, we consider the situation in which the rumor spread rate is greater than the rumor spread threshold (i.e., *λ* > *λ*_*c*_). In this situation, immunizing some of the individuals hinders the spread of the rumor. Since in homogeneous networks, we consider the effects of random immunization. If *p* represents the proportion of the nodes that are immunized, then under the assumption that we can ignore the time-lag effect, the initial condition for mean-field Eqs (1) to (4) becomes *I*(0) ≈ 1 − *p*, *S*(0) ≈ 0, *R*1(0) = 0 and *R*2(0) = *p*. With the new initial conditions and according to the previous algorithm, we can obtain:

$$\begin{array}{ccc} R1(t) & = & \left(-\gamma +\frac{\alpha \beta }{\theta +1}\right)I\left(t\right)+\frac{\alpha \left(\theta +1\right)p{\left(1-p\right)}^{\theta }+\alpha \beta {\left(1-p\right)}^{\theta +1}}{\theta (\theta +1)}I{\left(t\right)}^{-\theta }\\ & & -\frac{\delta }{\bar{k}}\ln I\left(t\right)+\gamma \left(1-p\right)-\frac{\alpha \beta (1-p)+\alpha p}{\theta }+\frac{\delta }{\bar{k}}\ln\left(1-p\right) \end{array}$$

$$\begin{array}{c} R2(t)=-\frac{\beta }{\theta +1}I\left(t\right)+\frac{\left(\theta +1\right)p{\left(1-p\right)}^{\theta }+\beta {\left(1-p\right)}^{\theta +1}}{\theta +1}I{\left(t\right)}^{-\theta } \end{array}$$

So:

$$\begin{array}{ccc} & & I=1-\left(-\gamma +\frac{\alpha \beta }{\theta +1}\right)I-\frac{\alpha \left(\theta +1\right)p{\left(1-p\right)}^{\theta }+\alpha \beta {\left(1-p\right)}^{\theta +1}}{\theta (\theta +1)}I^{-\theta }+\frac{\delta }{\bar{k}}\ln I-\gamma \left(1-p\right)\\ & \;\;\; & \;+\frac{\alpha \beta (1-p)+\alpha p}{\theta }-\frac{\delta }{\bar{k}}\ln\left(1-p\right)+\frac{\beta }{\theta +1}I-\frac{\left(\theta +1\right)p{\left(1-p\right)}^{\theta }+\beta {\left(1-p\right)}^{\theta +1}}{\theta +1}I^{-\theta }\\ & \Rightarrow & R=-\gamma p-\frac{\alpha \beta (1-p)+\alpha p}{\theta }-\frac{\beta -\alpha \beta }{\theta +1}+\frac{\delta }{\bar{k}}\ln\left(1-p\right)+\left(\gamma +\frac{\beta -\alpha \beta }{\theta +1}\right)R\\ & \;\;\; & \;+\frac{\left(\alpha +\theta \right)\left(\theta p+p+\beta -\beta p\right){\left(1-p\right)}^{\theta }}{\theta (\theta +1)}{\left(1-R\right)}^{-\theta }-\frac{\delta }{\bar{k}}\ln\left(1-R\right) \end{array}$$

Given:

$$\begin{array}{ccc} g(R) & = & R+\gamma p+\frac{\alpha \beta (1-p)+\alpha p}{\theta }+\frac{\beta -\alpha \beta }{\theta +1}-\frac{\delta }{\bar{k}}\ln\left(1-p\right)-\left(\gamma +\frac{\beta -\alpha \beta }{\theta +1}\right)R\\ & & \;-\frac{\left(\alpha +\theta \right)\left(\theta p+p+\beta -\beta p\right){\left(1-p\right)}^{\theta }}{\theta (\theta +1)}{\left(1-R\right)}^{-\theta }+\frac{\delta }{\bar{k}}\ln\left(1-R\right) \end{array}$$

Taking the derivative of *g*(*R*) with respect to *R*, we have:

$$\begin{array}{ccc} g\prime \left(R\right) & = & 1-\gamma -\frac{\beta -\alpha \beta }{\theta +1}-\frac{\left(\alpha +\theta \right)\left(\theta p+p+\beta -\beta p\right){\left(1-p\right)}^{\theta }}{\theta +1}{\left(1-R\right)}^{-\theta -1}-\frac{\delta }{\bar{k}}\frac{1}{1-R}\\ g\prime \prime \left(R\right) & = & -\left[\left(\alpha +\theta \right)\left(\theta p+p+\beta -\beta p\right){\left(1-p\right)}^{\theta }\right]{\left(1-R\right)}^{-\theta -2}-\frac{\delta }{\bar{k}}{\left(1-R\right)}^{-2}\\ ∵ & & 0<\beta <1,0\leq p\leq 1\\ ∴ & & g\prime \prime \left(R\right)<0 \end{array}$$

That is, *g*(*R*) is also a concave function on the interval [0, 1), and:

$$\begin{array}{ccc} g(p) & = & \left(1-\gamma -\frac{\beta -\alpha \beta }{\theta +1}\right)p-\frac{\left(\alpha +\theta \right)\left(\theta p+p+\beta -\beta p\right){\left(1-p\right)}^{\theta }}{\theta (\theta +1)}{\left(1-p\right)}^{-\theta }+\frac{\delta }{\bar{k}}\ln\left(1-p\right)\\ & & \;+\gamma p+\frac{\alpha \beta (1-p)+\alpha p}{\theta }+\frac{\beta -\alpha \beta }{\theta +1}-\frac{\delta }{\bar{k}}\ln\left(1-p\right)=0 \end{array}$$

$$\begin{array}{ccc} g\prime \left(p\right) & = & 1-\gamma -\frac{\beta -\alpha \beta }{\theta +1}-\frac{\left(\alpha +\theta \right)\left(\theta p+p+\beta -\beta p\right){\left(1-p\right)}^{\theta }}{\theta +1}{\left(1-p\right)}^{-\theta -1}-\frac{\delta }{\bar{k}}{\left(1-p\right)}^{-1}\\ & = & \frac{\lambda \bar{k}-\left(\lambda +\alpha +\theta \right)\bar{k}p-\delta }{\left(1-p\right)\bar{k}} \end{array}$$

$$\begin{array}{ccc} \lim_{R\to 1-}g\left(R\right) & = & \lim_{R\to 1-}[\left(1-\gamma -\frac{\beta -\alpha \beta }{\theta +1}\right)R-\frac{\left(\alpha +\theta \right)\left(\theta p+p+\beta -\beta p\right){\left(1-p\right)}^{\theta }}{\theta (\theta +1)}{\left(1-R\right)}^{-\theta }\\ & & \;+\frac{\delta }{\bar{k}}\ln\left(1-R\right)+\gamma p+\frac{\alpha \beta (1-p)+\alpha p}{\theta }+\frac{\beta -\alpha \beta }{\theta +1}-\frac{\delta }{\bar{k}}\ln\left(1-p\right)]\\ & = & 1-\gamma +\gamma p+\frac{\alpha \beta (1-p)+\alpha p}{\theta }-\frac{\delta }{\bar{k}}\ln\left(1-p\right)-\infty -\infty \\ & = & -\infty <0 \end{array}$$

**Fig 3:**
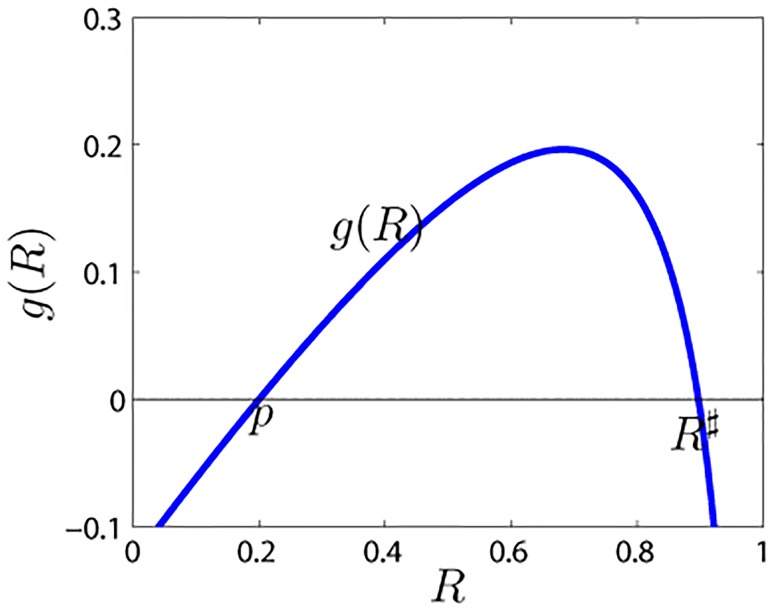
Shape of the function *g*(*R*). The parameters are $\beta =0.03,\gamma =0.15,\theta =0.45,\alpha =0.25,\delta =0.35,\bar{k}=10,$ and *p* = 0.2.

So, considering *g*′(*p*) > 0 (i.e., $p<\frac{\lambda \bar{k}-\delta }{(\lambda +\alpha +\theta )\bar{k}}$), there exists an *R* ^♯^ ∈ (*p*, 1) satisfying *g*(*R* ^♯^) = 0 (Fig 3). In other words, we obtain the immunization threshold $p_{c}=\frac{\lambda \bar{k}-\delta }{(\lambda +\alpha +\theta )\bar{k}}$ in the modified rumor model. When *p* > *p*_*c*_, *g*′(*p*) < 0, and there is only one intersection of *g*(*R*) with the *x*-axis, thus *R* = *p* = *R*1 + *R*2. Considering that *R*1(0) = 0 and *R*2(0) = *p*, then *R*1 = *R*1(∞) = 0, *R*(∞) = *R*2(∞) = *R*2(0). This shows that the rumor is no longer propagated.

**Fig 4:**
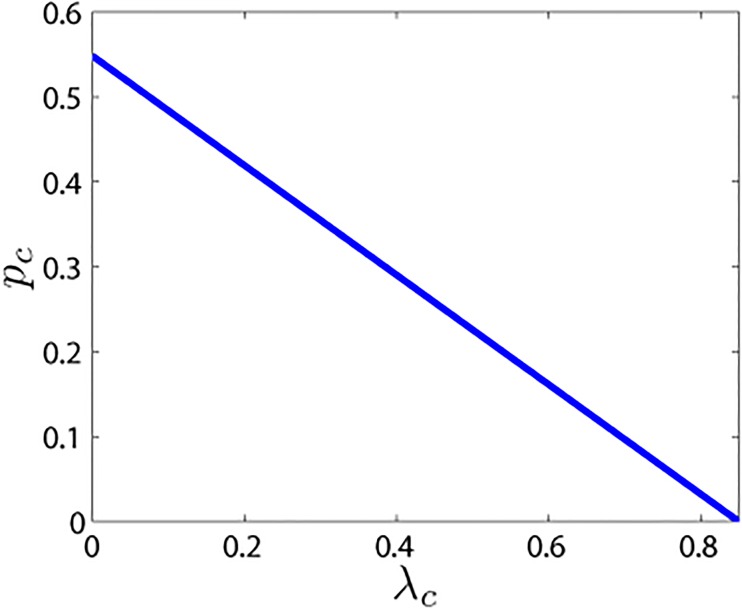
The relationship between the spread threshold (*λ*_*c*_) and the immunization threshold (*p*_*c*_). The parameters are *λ* = 0.85, *θ* = 0.45, *α* = 0.25.

Moreover, $\lambda_{c}=\frac{\delta }{\bar{k}}$; that is, $\delta =\lambda_{c}\bar{k}$. Because $p_{c}=\frac{\lambda \bar{k}-\delta }{(\lambda +\alpha +\theta )\bar{k}}$, $p_{c}=\frac{\lambda -\lambda_{c}}{\lambda +\alpha +\theta }$. This means that the immunization threshold *p*_*c*_ varies inversely with the spreading threshold *λ*_*c*_ (Fig 4). As *λ*_*c*_ increases, it becomes more difficult for the rumor to spread and fewer nodes must be immunized, making it more feasible to prevent the rumor from spreading.

## Numerical simulations

**Fig 5:**
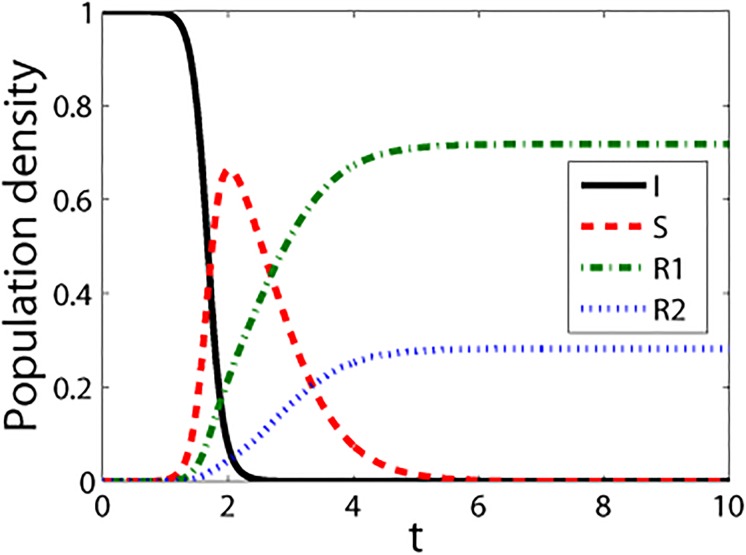
Densities of the four groups in the rumor model over time(*t*). The parameters are *λ* = 0.85, *β* = 0.03, *γ* = 0.12, *α* = *θ* = 0.25, *δ* = 0.35, and $\bar{k}=10$.

We used simulations to investigate the properties of the modified rumor-spread model. Moreno et al. [17] compared the stochastic numerical approach that is based on the numerical solution of the mean-field equations with the Monte Carlo simulation method describing the rumor spreading process. They found that the difference between the two methods is less than 1.4%, which indicates the reliability of the stochastic numerical approach. Moreover, they found the stochastic numerical approach is much faster than the Monte Carlo method. Therefore, we also apply the stochastic numerical approach to simulations. Specifically, we use the Runge-Kutta method to solve differential Eqs (1)–(4). We assume that the size of the homogeneous network is *N* = 10^6^ and that there is only one spreader as the initial condition, thus $S(0)=\frac{1}{10^{6}},I(0)=\frac{10^{6}-1}{10^{6}}$ and *R*1(0) = *R*2(0) = 0. Fig 5 illustrates the general trends for the four groups of individuals over time based on the modified model. Fig 5 shows that the number of spreaders initially increases, reaches a peak, and then decreases to 0, which means that the rumor no longer spreads. During this process, the number of ignorants steadily decreases, while the numbers of R1 stiflers and R2 stiflers steadily increase until they reach an equilibrium state.

In our model, there are 1 parameter about the network structure characteristic and 6 probability parameters about state transformation among different classification of the crowd. The average degree $\bar{k}$ is the key parameter for describing the homogeneous network characteristic. The more one person contacts with other people, the greater the $\bar{k}$ is. The transformation probability values can be understood from two aspects: one is from the individual level, the other is from the group level. Let the parameter *β* to illustrate. We know that *β* is the transformation probability from ignorant state to R2 stifler because the rumor was seen through directly by the ignorant individuals. From the individual point of view, an ignorant with more knowledge and more reason, is transformed into R2 stifler with a higher probability, that is, the value of *β* is greater. From the group point of view, the ignorants endowed with immunity to rumors are latent rational agents, and their proportion in the crowd can be embodied in the probability *β*. Generally, the proportion of latent rational agents account for the population is 0.001%–3%, then the range of parameter *β* is 0.001%–3%, and this proportion can certainly be improved through better education. Spreading rate *λ* is not only related to the characteristics of people, but also related to the importance and the attraction of the rumor. The closer the relationship between the rumor and people, the greater the value of *λ*. In fact, when *β* and *λ* are fixed, the probability *γ* is set because of *γ* = 1 − *λ* − *β*. The more unimportance and more boring the rumor is, the greater the parameter *γ* is. Besides the trait of the spreader, parameters *θ* and *α* are also related to the feature of the R2 stifler. If the R2 stifler is a convincing authority person (like scientist), then probabilities *θ* and *α* would become bigger, and even *θ* is likely to be greater than *α*. Forgetting rate *δ* should be in accordance with the law of people’s forgetfulness, that is gradually smaller with the passage of time. However, in this article, we defined it as constant for convenience of calculations.

We can find some insights that the significant parameters impact on the rumor spreading by numerical simulations. In the steady state, the sum of R1 stiflers and R2 stiflers changes little with the changes in these parameters. This reflects the parameters have less effect on the total size of the rumor R. But the final values of R1 stiflers and R2 stiflers always show the opposite trend with the change of every parameter above. For instance, the final value of the R1 stiflers decreases, whereas the final value of the R2 stiflers increases with the increase of average degree $\bar{k}$. R2 stiflers represent the people who see the falsity of the rumor and oppose its spread. R2 stiflers are key to terminate the rumor spreading. Therefore, the sovereign should pay more attention to the R2 stiflers. The parameter *β* plays a positive role for the increase of R2 stiflers. Better education can increase the parameter *β*. If R2 stiflers (e.g. government officials) have more powerful credibility, that is the greater *θ*, more spreaders will transfer to R2 stiflers. Furthermore, the peak of rumor spreading will appear early with the increase of the average degree $\bar{k}$, so the sovereign should take measures (such as immunization) to prevent and control the spread of the rumor as soon as possible in the closely-knit networks. The detailed effect of these parameters on the rumor spreading can be found in the Supporting Information (S1 Fig, S2 Fig, S3 Fig).

**Fig 6:**
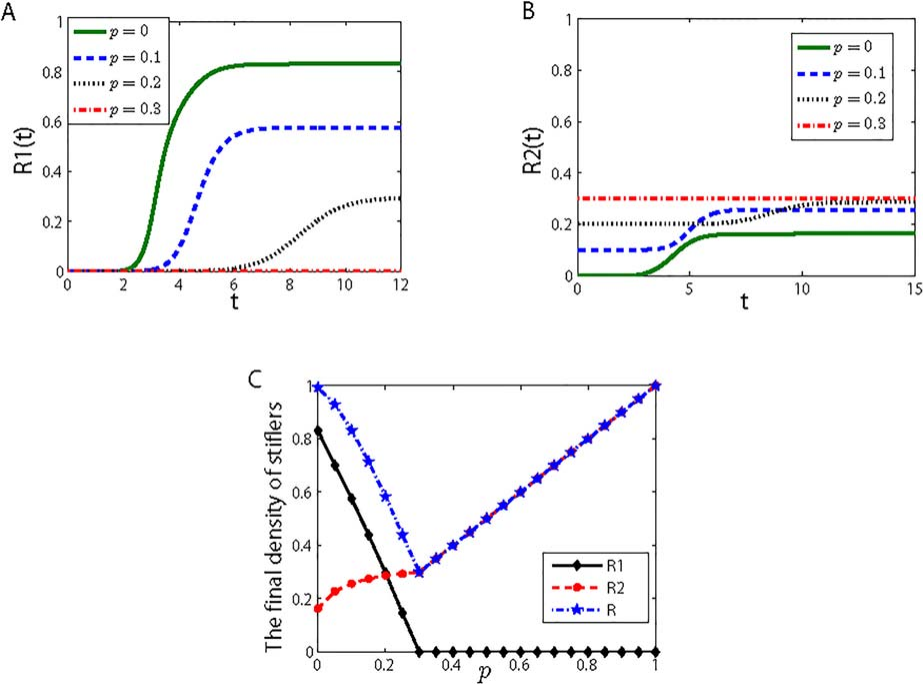
Nodes immunization effects on the prevention and control of rumors-spread. A) Changes in the density of R1 stiflers over time (*t*) as a function of the immunization rate *p*. B) Changes in the density of R2 stiflers over time (*t*) as a function of the immunization rate *p*. C) Changes in the final value of the R1 and R2 stiflers, and the final size of the rumor (*R*) as a function of the proportion of the population that is immunized *p*. The values of the model parameters are *λ* = 0.45, *β* = 0.02, *γ* = 0.53, *α* = 0.45, *θ* = 0.50, *δ* = 0.35, and $\bar{k}=10$.

Fig 6 shows the role of random immunization against the rumor spreading. A-B: The densities of R1 stifler and R2 stifler show different patterns as a function of time (*t*) with the variable immunization rate *p*. From Fig 6A, according to the increase of immunization rate *p*, the final value of R1 stifler reduces. When the immunized population covers 30%, the final value of R1 stifler drops more than 80% and equals to 0. As in Fig 6B, when immunization rate *p* increases, the final value of R2 stifler increases, due to that we defined the immunized nodes as R2 stiflers. When *p* equals to 0, 0.1 or 0.2, the gradual changes of R2 stifler all show an increase process because of the increase of R2 stifler from the rumor spreading. When *p* = 0.3, the density of R2 stifler remains the same level as time goes by, in which condition the immunization rate is more than the immunization threshold and the rumor fail to spread. Fig 6C more apparently shows the changes of stiflers due to the variation of immunization rate, in which the points indicate the final densities of stiflers when *p* varies from 0 to 1. As *p* increases, the final value for the R1 stiflers decreases, eventually reaching 0 at the immunization threshold *p*_*c*_, which is approximately 0.3 under these simulation conditions. This agrees well with the calculated results obtained from the analysis of *p*_*c*_ in the previous section. Fig 6C also shows that the final value of R2 stiflers increases continuously: it rises slowly before it reaches *p*_*c*_, then increases at a faster but stable rate. When *p* = 0, indicating no immunization in the system, the final size of the rumor (*R*) almost reaches 1. As *p* increases, *R* first decreases until it reaches its minimum value at point *p*_*c*_, which demonstrates the effectiveness of the immunization strategy, then subsequently increases parallel to the increase in the final value of R2 stiflers which means more people who know the rumor is a false information because of the immunization effect. When the immunization rate equals to the immunization threshold, the rumor fails to spread, then it is unnecessary to increase the immunization rate. Because immunization strategy is not free, and it needs material resources, excessive immunization is a waste of these resources. Furthermore, under the circumstance that the rumor spread failure, let more ignorants be immunized, that is, let more ignorants know the rumor is a false information, which waste the time and energy of ignorants. Hence, excessive immunization is also unnecessary. Therefore, inoculating a higher proportion of the population is not always the optimal solution, and it is important to determine the optimal transition point *p*_*c*_. Obtaining the value of immunization threshold *p*_*c*_ using our model means obtaining the most effective immunization rate to control spreading of the rumor.

## Conclusions

In this paper, we investigated the dynamics of a modified rumor-spread model that accounts for an immunization strategy that takes advantage of homogenous networks. Based on the SIR rumor-spread model, we divided the stiflers into two groups with different characteristics and thereby changed the mechanism responsible for termination of the rumor’s spread. We then established a modified rumor-spread model and derived the mean-field equations for the model for a homogeneous network. We determined the spreading threshold *λ*_*c*_ by analyzing the mean-field equations at the steady-state. Furthermore, we investigated the effects of random immunization and obtained the immunization threshold, *p*_*c*_, that provides optimal reduction of the rumor’s spread. There was an inverse relationship between the immunization rate and the spreading threshold. Numerical simulations illustrated the dynamics of the modified rumor model.

If the rumor spreading probability is less than or equal to the spreading threshold (i.e., *λ* ≤ *λ*_*c*_), then the rumor will not spread, so reducing the rumor’s probability of spreading or increasing the rumor’s spreading threshold is an effective way to prevent rumor transmission. In contrast, when the spreading probability is greater than the spreading threshold (i.e., *λ* > *λ*_*c*_), the rumor may be propagating, and applying the immunization strategy will be required to effectively prevent the spread of the rumor. However, this does not mean that increasing the number of immunized nodes will always be better. The optimal solution is to choose an immunization rate equal to the immunization threshold, at which point the rumor will not spread and the number of people who learn about the false rumor will reach its minimum. Apart from immunization strategy, we discovered by simulations that to increase the proportion of rational agents (the greater *β*) could probably prevent rumor propagation. Education can be an important tool, as to teach more people how to recognize the rumor, more people would become rational agents. Furthermore, when dispelling the rumor, the R2 stiflers, who have more powerful credibility (the greater *θ* and *α*), can be more effective to prevent rumor propagation.

Rumor spreading is a complex process, of which our proposed model will be further improved from the following prospectives. First, the values of all these parameters in our model were complicated to be quantified, presumably associated with a couple of important elements. However, very few quantifications of the parameters have been proposed in publications on rumor spreading so far. In reality, the actual values vary a lot in different rumor events. Second, our model was constructed based on homogenous networks. However, to calculate the immunization threshold of the larger number of heterogeneous networks in reality requires further investigation. Finally, we have ignored the time-lag effect in our model, while in reality there must be time delay in node immunization. Therefore, it’ll be inevitable to take into account the effect of time delay during rumor prevention.

## Supporting Information

S1 Fig Changes in the densities of the four kinds of people over time (*t*) as a function of the average network degree ($\bar{k}$). A)Ignorants; B) Spreaders; C) R1 stiflers; and D) R2 stiflers. The other parameters are *λ* = 0.85, *β* = 0.03, *γ* = 0.12, *α* = *θ* = 0.25, and *δ* = 0.35.
(TIF)

S2 Fig Changes in the densities of R1 stiflers and R2 stiflers over time (*t*) as a function of the parameters *λ*, *β* and *λ*, *γ*. A) R1 stiflers changing with *λ* and *β*. B) R2 stiflers changing with *λ* and *β*. In Figures A and B, the other parameters are $\gamma =0.30,\alpha =\theta =0.25,\delta =0.35,\bar{k}=10$. C) R1 stiflers changing with *λ* and *γ*. D) R2 stiflers changing with *λ* and *γ*. In Figures C and D, the other parameters are $\beta =0.02,\alpha =\theta =0.25,\delta =0.35,\bar{k}=10.$
(TIF)

S3 Fig Changes in the densities of R1 stiflers and R2 stiflers over time (*t*) as a function of the parameters *δ* and *α*, *θ*. A) R1 stiflers changing with *δ*. B) R2 stiflers changing with *δ*. In Figures A and B, the other parameters are $\lambda =0.50,\beta =0.02,\gamma =0.48,\alpha =\theta =0.25,\bar{k}=10$. C) R1 stiflers changing with *α* and *θ*. D) R2 stiflers changing with *α* and *θ*. In Figures C and D, the other parameters are $\lambda =0.50,\beta =0.02,\gamma =0.48,\delta =0.30,\bar{k}=10.$
(TIF)

S1 File Supplementary information: the sensitivity analysis of parameter $\bar{k}$. (DOCX)

S2 File Supplementary information: the sensitivity analysis of parameters *λ*, *β* and *γ*. (DOCX)

S3 File Supplementary information: the sensitivity analysis of parameter *δ*, *α* and *θ*. (DOCX)
