# Supplementary figures and images for: Immunization against the Spread of Rumors in Homogenous Networks
Source: PLoS One. 2015 May 1;10(5):e0124978. doi: 10.1371/journal.pone.0124978 (PMC4416730; doi:10.1371/journal.pone.0124978)

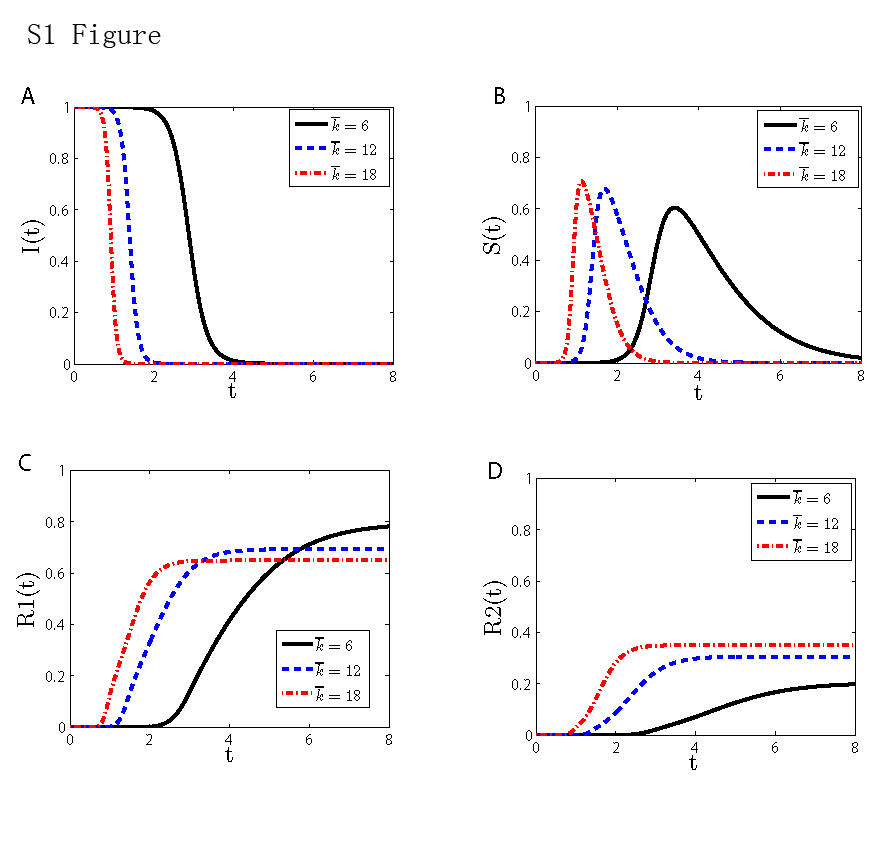

Supplement: S1 Fig — A)Ignorants; B) Spreaders; C) R1 stiflers; and D) R2 stiflers. The other parameters are λ = 0.85, β = 0.03, γ = 0.12, α = θ = 0.25, and δ = 0.35. (TIF) [file pone.0124978.s001.tif]

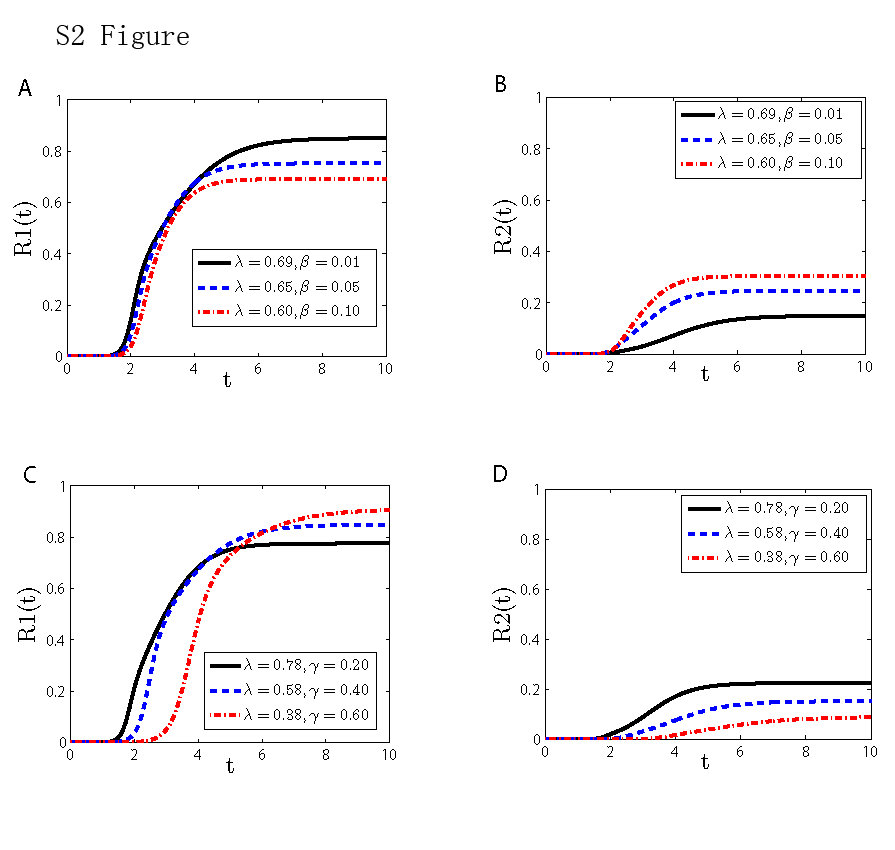

Supplement: S2 Fig — A) R1 stiflers changing with λ and β. B) R2 stiflers changing with λ and β. In Figures A and B, the other parameters are γ=0.30,α=θ=0.25,δ=0.35,k¯=10. C) R1 stiflers changing with λ and γ. D) R2 stiflers changing with λ and γ. In Figures C and D, the other parameters are β=0.02,α=θ=0.25,δ=0.35,k¯=10. (TIF) [file pone.0124978.s002.tif]

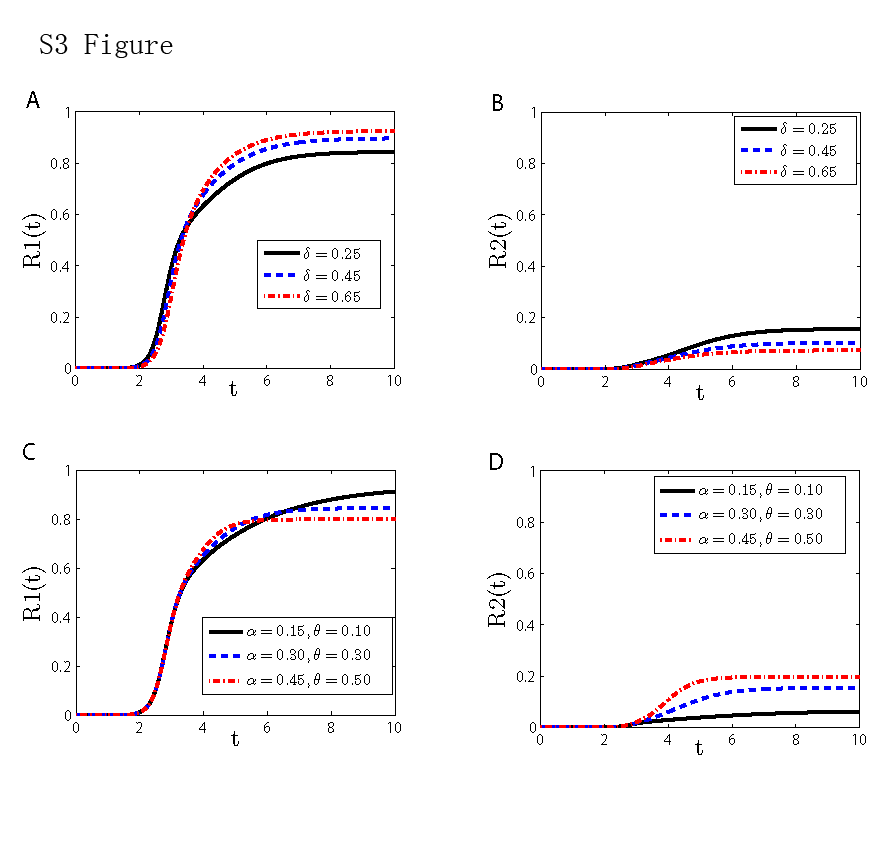

Supplement: S3 Fig — A) R1 stiflers changing with δ. B) R2 stiflers changing with δ. In Figures A and B, the other parameters are λ=0.50,β=0.02,γ=0.48,α=θ=0.25,k¯=10. C) R1 stiflers changing with α and θ. D) R2 stiflers changing with α and θ. In Figures C and D, the other parameters are λ=0.50,β=0.02,γ=0.48,δ=0.30,k¯=10. (TIF) [file pone.0124978.s003.tif]
