# Supplementary material for: Immunization against the Spread of Rumors in Homogenous Networks
Source: PLoS One. 2015 May 1;10(5):e0124978. doi: 10.1371/journal.pone.0124978 (PMC4416730; doi:10.1371/journal.pone.0124978)
Supplement: S1 File — (DOCX) [file pone.0124978.s004.docx]

**S1 File. Supplementary information:** **the sensitivity analysis of parameter** $\bar{\boldsymbol{k}}$**.**

S1 Figure illustrates how the densities of the four groups of people vary over time in response to changes in the average degree, $\bar{k}$. These results show that the bigger the average degree, the less time it takes for the system to reach the equilibrium state in which spreading of the rumor stops. Figure S1B shows the population of the spreaders reaches a larger peak value as $\bar{k}$ increases, which demonstrates that the maximum rumor influence is positively related to the average degree of the network. Moreover, Figure S1C and Figure S1D show that the final value of the R1 stiflers decreases with increasing average degree, whereas the final value of the R2 stiflers increases for the same change in the average degree. The final size of the rumor, *R*, which equals 1 minus *I* (where *R*= *R1* + *R2*), is very little change with increasing$\bar{k},$ obtained from Figure S1A. However, the number of R1 stiflers decreases, which reflects the fact that in more highly connected networks, people are more likely to recognize the rumor and learn the truth.
