# Supplementary material for: Immunization against the Spread of Rumors in Homogenous Networks
Source: PLoS One. 2015 May 1;10(5):e0124978. doi: 10.1371/journal.pone.0124978 (PMC4416730; doi:10.1371/journal.pone.0124978)
Supplement: S2 File — (DOCX) [file pone.0124978.s005.docx]

**S2 File. Supplementary information: the sensitivity analysis of parameters λ, β and γ.**

S2 Figure illustrates the changes of R1stifler and R2 stifler according to the variation of these parameters (λ, β and γ) associated with ignorants. A-B: With the constant value of γ and three different series of λ and β, the densities of R1 stifler and R2 stifler show different plots as the function of time (t), separately. Along with the decrease of λ and increase of β, the final value of R1 stifler decreases while R2 stifler increases and the time of rumor ending is reduced. That means the rumor can be prevented from spreading more efficiently with the more rational people in the population, which is identical with the reality. C-D: With the constant value of β, three different series of λ and γ (to weaken the importance and attractiveness of the rumor), R1 stifler and R2 stifler vary as the function of time (t). Along with the decrease of λ and increase of γ, the final value of R1 stifler increases while R2 stifler decreases. As parameter γ associates with ignorants transferring into R1 stiflers directly, whereas most of R2 stiflers should originate from spreaders, and smaller λ means less spreaders and less R2 stiflers.
