# Supplementary material for: Immunization against the Spread of Rumors in Homogenous Networks
Source: PLoS One. 2015 May 1;10(5):e0124978. doi: 10.1371/journal.pone.0124978 (PMC4416730; doi:10.1371/journal.pone.0124978)
Supplement: S3 File — (DOCX) [file pone.0124978.s006.docx]

**S3 File. Supplementary information: the sensitivity analysis of parameters δ, α and θ.**

S3 Figure displays the effects of R1 stifler and R2 stifler according the variation of parameters (δ, α and θ) associated with spreaders. A-B: With variable forgetting rate δ, R1 stifler and R2 stifler show different patterns as a function of time (t). Along with the increase of δ, the final value of R1 stifler increases while R2 stifler's final value reduces, most likely because that people who forget the rumor or who have no interest in the rumor are not the ones seeing through the rumor. C-D: As α and θ vary in the same direction (to enhance the reliability of rumor-recognizing people), R1 stifler and R2 stifler show different pattern as a function of time (t) separately, assuming the variation amplitude of θ is bigger than α. According the increase of α and θ, the final value of R1 stifler reduces while the final value of R2 stifler increases, which means the more reliability rumor-recognizing people have, the more people would be affected to see through the rumor.
